# Excellent Combination of Tensile ductility and strength due to nanotwinning and a biamodal structure in cryorolled austenitic stainless steel

**DOI:** 10.1038/s41598-019-57208-x

**Published:** 2020-01-15

**Authors:** G. Venkata Sarath Kumar, K. R. Mangipudi, G. V. S. Sastry, Lalit Kumar Singh, S. Dhanasekaran, K. Sivaprasad

**Affiliations:** 1 Advanced Materials Processing Laboratory, Department of Metallurgical and Materials Engineering, National Institute of Technology, Tiruchirappalli, 620015 Tamil Nadu India; 2 Technical Center, Ashokleyland, Chennai, 600103 Tamil Nadu India; 3 School of Minerals, Metallurgical and Materials Engineering, Indian Institute of Technology Bhubaneswar-752050, Arugul, Khurda, Odisha India; 4 Department of Metallurgical Engineering, Indian Institute of Technology (BHU), Varanasi, 221005 Uttar Pradesh India

**Keywords:** Metals and alloys, Mechanical properties, Mechanical engineering

## Abstract

Austenitic stainless steels are prominent materials for their superior corrosion resistance and a combination of strength and ductility. However, the relatively low yield strength limits its application in high strength structural applications. Cryorolling is one of the promising methods of enhancing the mechanical properties of sheet metals. In the present work, Cryorolling of UNS S31000 stainless steel resulted in five-fold enhancement in yield strength with a significant loss in ductility. However, flash annealing at 800 °C for 120 s could restore its ductility up to 50% of its original ductility. The enhancement in strength is attributed to the formation of deformation nano-twins during flash annealing along with a bimodal grain structure.

## Introduction

Austenitic stainless steel (UNS S31000) exhibits excellent high temperature properties along with good corrosion resistance. Hence, it is widely used in applications such as chemical, petroleum, and cement industries^1^. However, owing to its good corrosion resistance arising from the high Cr content and single-phase microstructure, it is also used for Cryogenic tanks of missiles, space vehicles etc^2^. UNS S31000 stainless steel sheet products are generally produced by hot rolling of slabs, and possess a relatively low yield strength of around 250–300 MPa. Hence, it is highly desirable to enhance their strength, but without sacrificing their corrosion resistance and ductility. Introducing high density of dislocations by subjecting the steel to large plastic deformation by cold working is the most frequently used technique for this type of single phase steels in order to enhance strength^3^. The high density of dislocations leads to the formation of fine dislocation cell structure, which enhances the yield strength significantly^4^. The observed increase in yield stress due to cold working is known to be resulting from several strengthening phenomena. Two dominant contributions have been suggested for single phase alloys, namely, (i) dislocation strengthening due to interaction of dislocations and/or dislocation-twin interactions, and (ii) strengthening due to the interaction of dislocations with sub-grain cell wall structure^5^. Dynamic recovery, a thermally activated process, has been reported to occur while rolling even at relatively low homologous temperatures limiting the maximum attainable strength^6^. Thus, rolling at Cryogenic temperatures is believed to suppress dynamic recovery to achieve high strength levels. Significant efforts have thus been devoted to study strengthening mechanisms in Cryorolled non-ferrous alloys^7^. Krishna *et al*. studied the effect of Cryorolling on the mechanical behaviour of AA5083 alloy^8^, while Naga Krishna *et al*. investigated the strengthening mechanisms in Cryorolled Al-Cu alloy^9^. However, only a limited number of studies exist in literature on strengthening of high Ni stainless steels through Cryorolling^10^. Rezaee *et al*. Investigated increase of strength by developing ultrafine grained microstructure produced by cold rolling of low nickel stainless steel followed by annealing at 850 °C^11^. Similarly, Cryorolling followed by annealing was performed on Fe 25% Cr and 20% Ni by Xiong *et al*. and reported significant improvement in mechanical properties with no major loss in ductility^12^. Cryorolling of interstitial free steel (IF Steel) has been carried out by Sharma *et al*. and achieved double the strength due to grain refinement^13^. Nakoda *et al*. have reported studies on deformation induced martensite formation in 316 stainless steel rolled at room temperature^14^. However, no studies could be found on comparison between Cryorolled and Room-Temperature rolled UNS S31000 and their effect on the strength and ductility. Hence, the present study investigates the effect of rolling at room temperature and Cryogenic temperatures on the mechanical properties of UNS S31000 stainless steel. Since an enhancement in the strength is often associated with a reduction in ductility, we have also investigated three levels of flash annealing to optimize the recovery of ductility.

## Experimental Methods

Hot rolled UNS S31000 plates with a thickness of 6 mm with a nominal a composition (wt. %) of 0.064% C, 0.5% Si, 0.86% Mn, 26.36% Cr, 18.31% Ni, 0.25% Mo and 0.17% Cu, 0.015% Nb, 0.011% Ti, 0.052% V and Fe (remaining) were subsequently subjected rolling at a constant rolling speed of 12.5 cm/s in a two-high rolling mill having a 30 cm roll dia. Two rolling temperatures were selected: ambient and Cryogenic temperatures. In both cases, plates were subjects to either 50% or 75% reduction in thickness. Just before commencing Cryorolling, the plates were held in a liquid nitrogen bath (LNB) for 35 min and were subjected rolling immediately after removing from the bath. For intermediate passes, the dipping time was approximately 3 min. The reduction in each pass was chosen to be 0.1 mm for both room temperature rolled (RT) and Cryorolled (Cryo) plates in order to reduce adiabatic heating. After rolling, the samples were subjected to flash annealing at 800 °C and 850 °C. At each temperature, annealing was performed on different sample at 60 s, 120 s, and 180 s. The samples were mechanically ground using 100, 1000 and 5000 grit papers, and then polished with 0.05 μm non-crystalline silica colloidal suspension. Microtexture measurements were carried out using a Zeiss Supra25 FEG SEM integrated with electron backscattered diffraction (EBSD) system (Oxford Instruments with Nordy’s Detector). The instrument was supported by Aztec software packages including HKL channel 5 used for EBSD analysis. A step size of 1.2 μm was fixed for the EBSD measurements. TEM examination was performed on thin samples prepared from ~200 μm thick slices which were cut using a slow speed saw followed by mechanical grinding down to ~100 μm thickness. Just prior to examination, samples were twin-jet electropolished with standard electrolyte (methanol 78%, lactic acid 10%, sulphuric acid 7%, nitric acid 3%, and hydrofluoric acid 2%) at −30 °C. The thin foils were examined in a FEI® Tecnai G2, 20 S-twin transmission electron microscope operated at 200 kV and equipped with an EDAX®. The X-ray diffraction (XRD) studies were carried out (Cu-Kα radiation) in a Rigaku® Ultima III XRD unit for phase analysis.

## Results

### Effect of rolling on the stress-strain response

**Figure 1:**
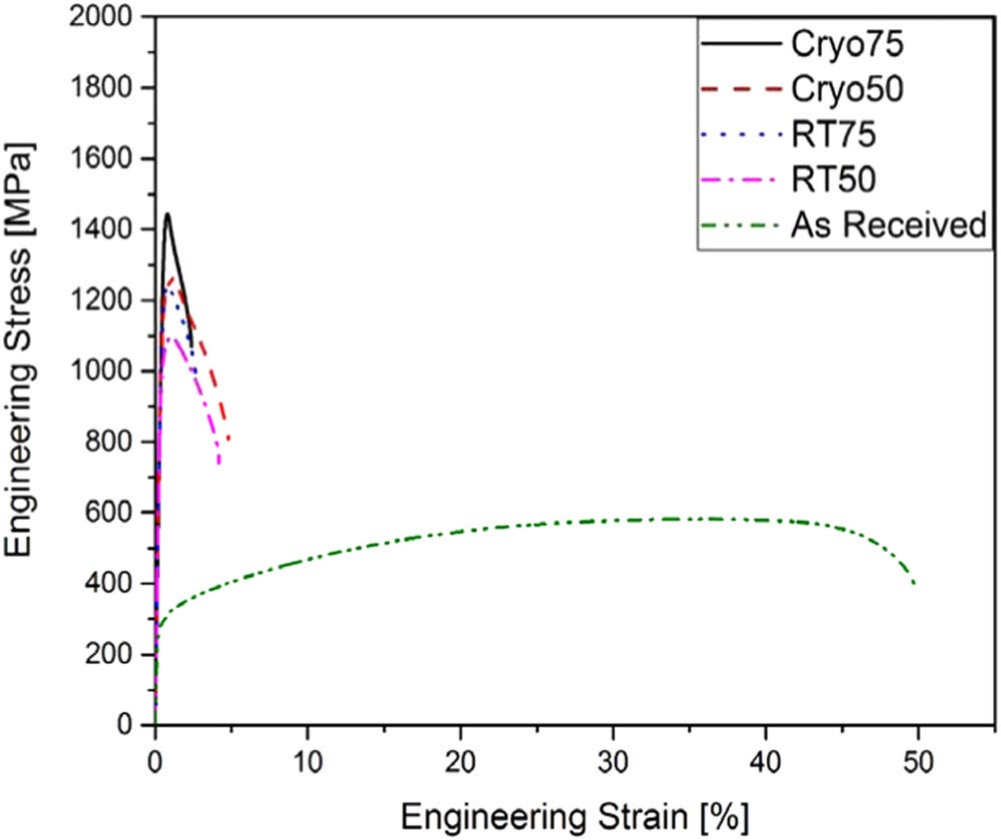
Effect of Cryo- and room temperature rolling on the stress-strain behaviour of UNS S31000 stainless steel.

The stress-strain response of the Cryorolled and room-temperature rolled samples at 50% and 75% thickness reductions are compared with that of as-received material in Fig. 1. The yield stress of the as-received sample is 280 MPa while its ultimate tensile stress is 581 MPa. The as-received material shows extensive strain hardening with an elongation to fracture reaching up to 50%. As expected, the two kinds of rolling methods increased the strength manifold, but with a severe drop in ductility.

The yield stress of the Cryorolled sample of 50% thickness reduction (Cryo50) has achieved a remarkable increase, as high as1186 MPa. However, the results on Cryo75 suggest that the rate of increase in the yield stress apparently decreases slightly with further deformation reaching only 1425 MPa at 75% thickness reduction. The room temperature rolling also produced similar strength values, but there was a slightly lower enhancement in the strength than that for a cryorolled sample. The yield stress at 50% and 75% reductions is 1045 MPa and 1237 MPa, respectively. The stress-strain curves of all the rolled samples exhibit a much lower strain hardening capacity in comparison to the as-received samples (Fig. 1).

### Effect of flash annealing on the mechanical properties

**Figure 2:**
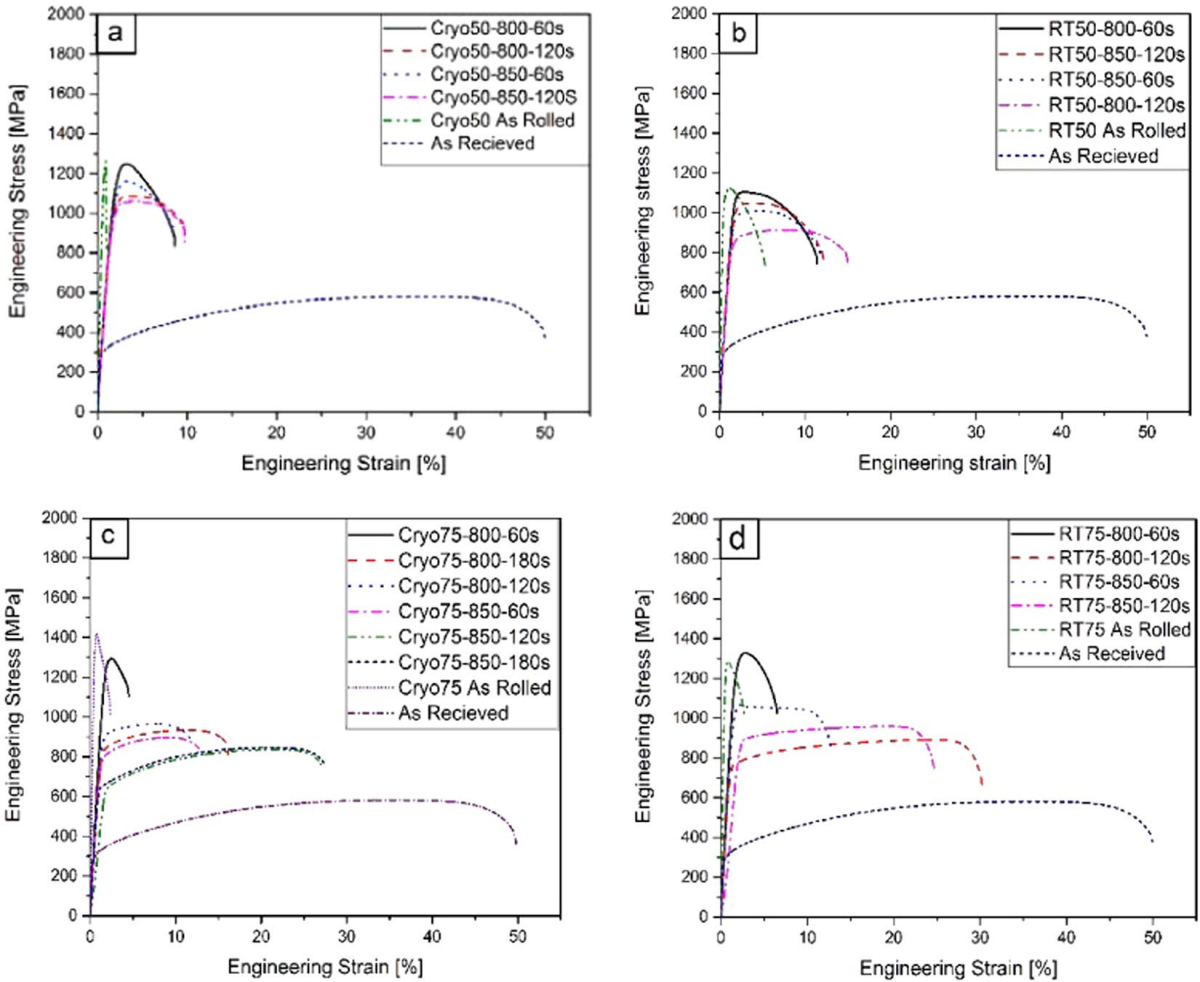
Stress-strains curves of rolled UNS S31000 stainless steel by Cryo- and room temperature rolling to 50% and 75% thickness reduction. (Refer to online version for colour reference).

Flash annealing after rolling leads to a decrease in the strength while simultaneously recovering some of the ductility (Fig. 2). The amount of decrease in the strength after the flash annealing is positively correlated with the annealing temperature and time. For example, after annealing at 800 °C for 60 s, the yield stress of the Cryo75 sample has decreased from $5{\sigma }_{ys}^{AR}$ to $4{\sigma }_{ys}^{AR}$, while for 120 s, it decreased to $3.1{\sigma }_{ys}^{AR}$, and to $3{\sigma }_{ys}^{AR}$ at 180 s. However, at 850 °C there is a drastic decrease in the yield stress to $2.8{\sigma }_{ys}^{AR}$ in the first 60 s of annealing itself. With further annealing time up to 180 s, the strength reduction appears to be highest at this temperature (Fig. 2(b)). The trends are qualitatively similar within other sets of samples (i.e., Cryo50, RT50, and RT75), although the effect is the strongest in Cryo75 when annealed at 800 °C. The yield stress under various annealing and rolling conditions is presented in (Fig. 2).

### Microstructural characterization

**Figure 3:**
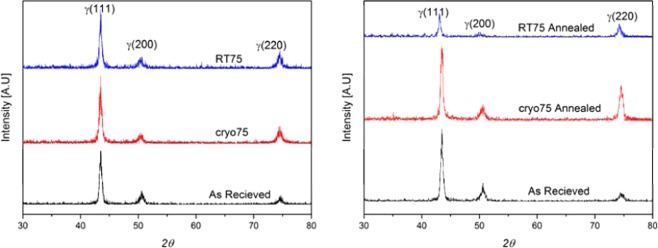
X-Ray diffraction patterns; (**a**) Rolled samples and (**b**) Rolled and annealed samples.

The UNS S31000 stainless steel is austenitic in the as-received condition which is produced by hot rolling of continuously cast slabs. To identify the phases present under various combinations of as-received, Cryorolled, RT rolled and annealed combinations, X-ray diffraction has been performed on the samples (Fig. 3). The X-ray diffraction patterns of all the above conditions indicate diffraction peaks corresponding to only the FCC phase, suggesting the presence of only austenite. This indicates that no major observable phase transformations have occurred within the resolution of the XRD during plastic deformation, even in the sample rolled at sub-zero temperature.

**Figure 4:**
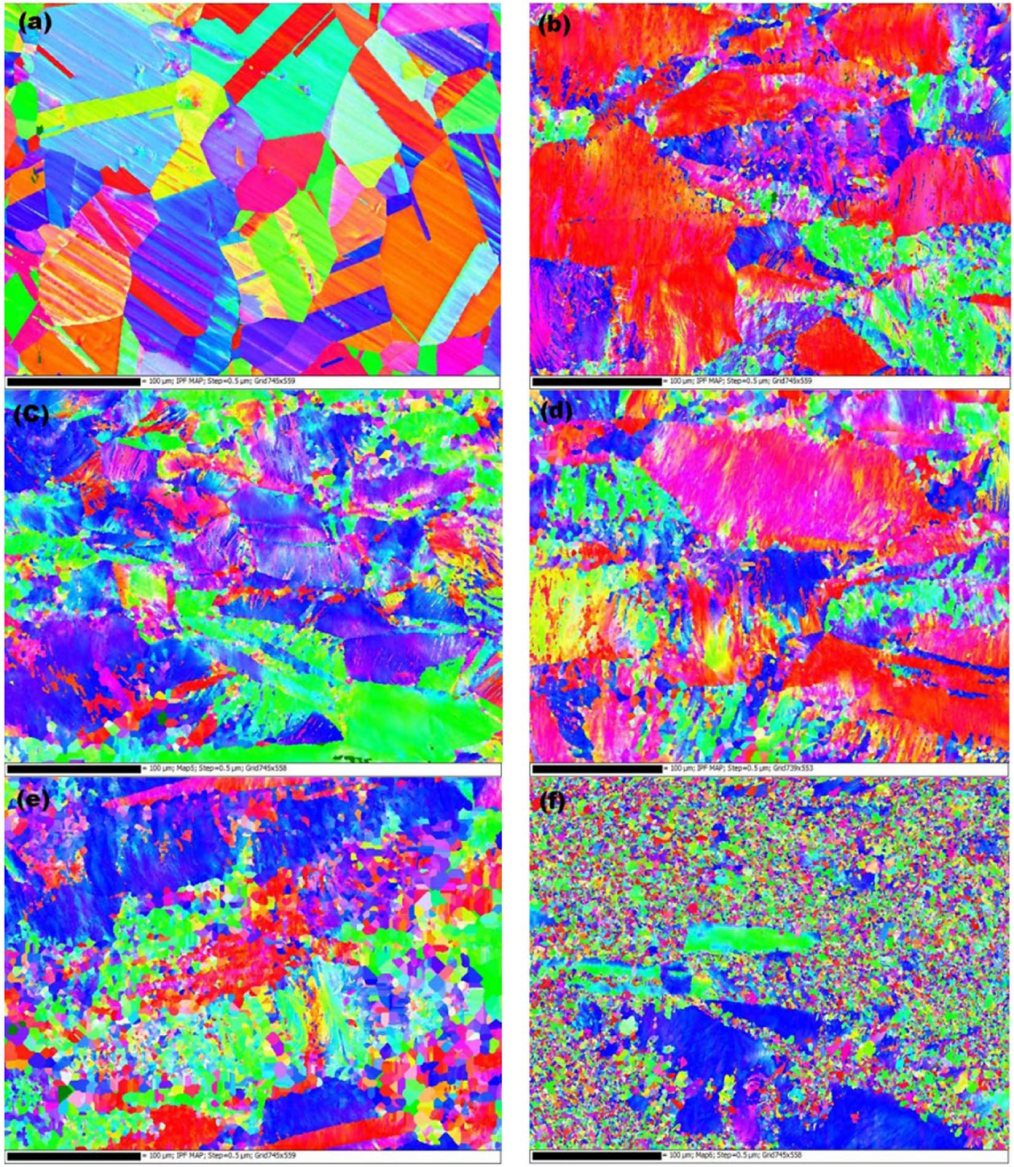
Inverse pole figure maps; (**a**) as received, (**b**) RT50, (**c**) Cryo50, (**d**) Cryo50 Annealed (**e**) Cryo75 and (**f**) Cryo75 Annealed conditions comparing grain size and crystallographic orientation.

EBSD analysis has been carried out to investigate the effect of the degree of deformation and temperature on the grain size distribution (Fig. 4). The as-received sample shows large twinned grains of the order of 100 μm in width, with a narrow grain size distribution (Fig. 4(a)). The annealing twins are about 20 μm in width. The RT50 (Fig. 4(b)) and Cryo50 (Fig. 4(c)) samples show highly strained regions due to large deformations that lead to the formation of ultra-fine grains with smooth crystallographic orientation gradients inside large grains. With increased deformation, the Cryo75 sample (Fig. 4(e)) forms clusters of well-defined fine recrystallized grains, which presumably would have formed when the sample is brought to room temperature after rolling. Similarly, annealing of Cryo50 sample also leads to recrystallization of highly strained regions of the grains. In contrast, the annealed Cryo75 sample shows extensive recrystallization with numerous ultrafine grains along with few larger grains as well evidencing a bimodal grain structure (Fig. 4(f)).

**Figure 5:**
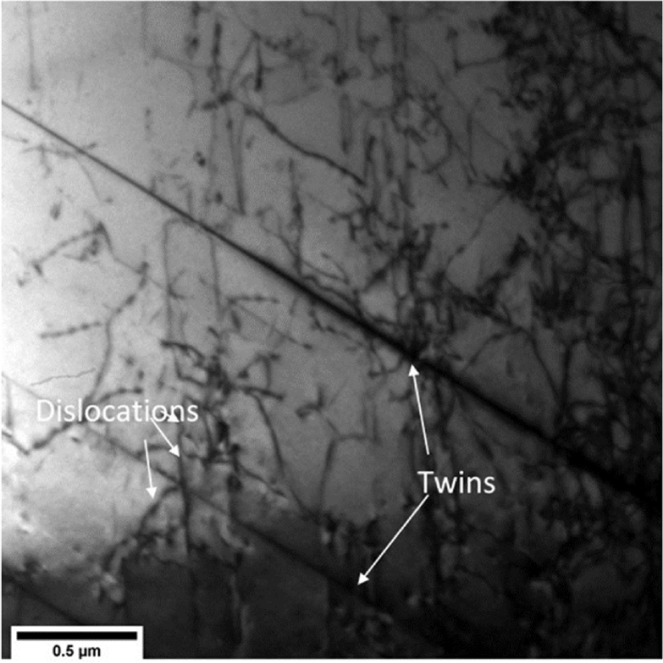
TEM micrograph of as-received sample showing relatively low dislocation density and widely spaced twins.

**Figure 6:**
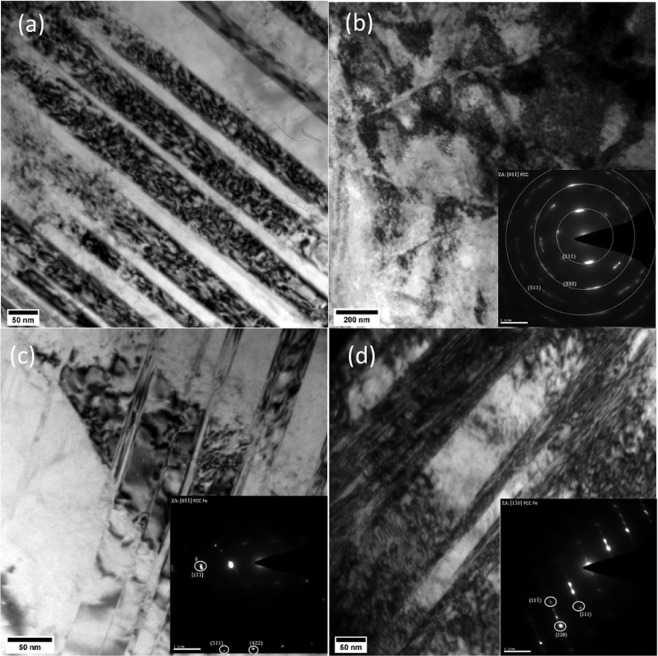
TEM micrographs of (**a**,**b**) Cryorolled 50%, (**c**) 75% thickness reduction and (**d**) Room temperature rolled sample of 50% thickness reduction.

**Figure 7:**
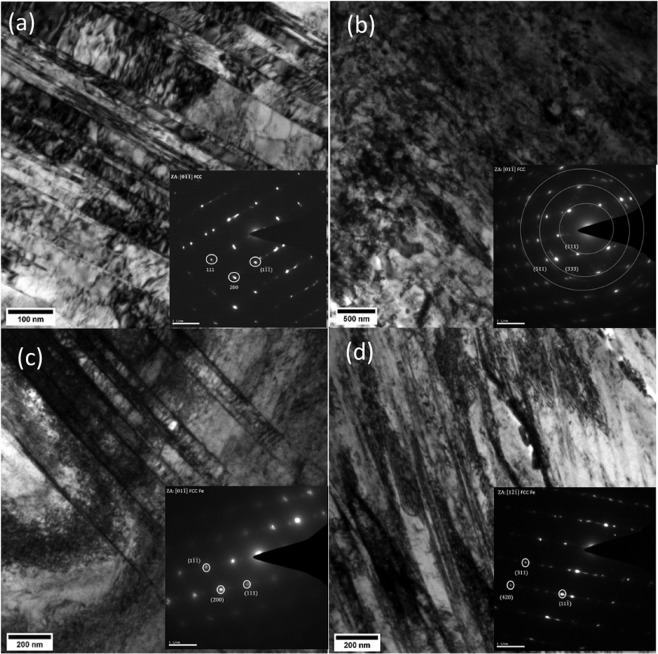
TEM micrographs of (**a**) Cryo50 annealed, (**b**) Cryo75 annealed, (**c**) RT50 annealed and (**d**) RT75 annealed.

The deformation mechanisms have been investigated using TEM after rolling and/or annealing. The as-received samples are characterized by relatively few twins and low dislocation density. TEM micrographs (Fig. 5) show that several dislocations move on intersecting slip planes and form entangled structures by interacting with other dislocations and twins. As expected, the dislocation density increases significantly for both Cryo and RT samples for both the 50% and 75% thickness reductions (Fig. 6). Nano-twins are observed on a large scale in these samples forming twin-matrix lamellar structure, see (a), (c), and (d) of (Fig. 6). High density dislocation cell structure can be seen inside these lamellae (Fig. 6(a)). Formation of a cross-hatched structure due to intersecting nano-twins of two variants is also observed in the Cryo50 sample (Fig. 6(b)). Due to large deformations, a highly dense dislocation structure forms within this intersecting structure and shows a tendency for the formation of ultra-fine grains, as suggested by the diffraction pattern shown in the inset of (Fig. 6(b)). Similar behaviour is also seen in Cryo75 and RT75.

(Figure 7) shows the TEM micrographs of the Cryorolled and RT rolled samples subjected to post-rolling flash annealing process. A comparison of Cryo50 samples before and after annealing (Figs. 6(a) and 7(a)) shows that the dislocation density within the twins decreases slightly, despite the presence of dislocations in both the twin-matrix lamellae. A very notable change may be the realignment of the dislocations. The ordered dislocation arrays on parallel slip planes with a characteristic spacing can be seen within a twin/matrix lamella (Fig. 7 (a)). This twin-matrix lamellar structure tends to break down after flash annealing of the Cryo75 samples (Fig. 7(b)). Additionally, some carbide nanoparticles have also been found in the Cryo75 annealed sample. In contrast to the Cryo50 annealed specimen, the RT50 annealed specimen shows a clear breakdown of the lamellar structure (Fig. 7(d)), although some twins with rearranged dislocation arrays similar to Cryo50 annealed sample could still be found in some regions (Fig. 7(c)). A large number of nano-twins are observed in the case of Cryorolled conditions than that of RT rolled samples. The nano-twin spacing (calculated from TEM images) is observed to be very small in the case of Cryorolled samples as shown in Table 1. The twin spacing is observed to increase during annealing in RT50 sample, whereas no major variation in twin spacing could be observed in the Cryo50 condition.

**Table 1:** Average twin spacing in different treated conditions of treatment of UNS S31000.

Condition	Cryo50	Cryo50 + Annealed	RT50	RT50 + Annealed
Avg. twin spacing(nm)	24	25	39	45

## Discussion

The room temperature and cryogenic conditions both offered a very significant increase in strength (four-fold and five-fold, respectively). A four-fold increase in the strength after room temperature rolling suggests that the primary strengthening mechanism is related to the introduction of high density of dislocations and twins as observed in the TEM images. The enhancement in the strength from the as received condition to rolled condition is suggested to be having contributions from (i) dislocation strengthening and (ii) grain boundary strengthening, Deformation substructures, such as dislocation cells, planar dislocations, deformation twins, deformation bands, and stacking faults have been observed in both the rolled conditions. The formation of deformation substructures critically depends on the stacking fault energy of the alloy. A low stacking fault energy (SFE) generally favours splitting of the partial dislocations and formation of large number of stacking faults^15^. This decreases dislocation mobility since cross slip becomes more difficult with widely spaced partials. This results in a relatively homogeneous dislocation distribution and decreased tendency for the formation of dislocation cell structure. A low SFE prefers deformation twinning at large grain sizes. Thus, for low SFE planar dislocation structures and deformation twins are formed, whereas high SFEs result in the formation of dislocation cell structures. The SFE of UNS S31000 is estimated to be between 33–80 mJ/m^2^ according different empirical expressions suggested in the literature^16–22^. Such a low SFE suggests that deformation twins as observed in the TEM images. It has also been reported that SFE increases with increasing deformation temperature^23^. Thus, under Cryorolling conditions a further decrease in SFE value is expected that may lead to additional twinning (see Fig. 6(a,d)). A low SFE also encourages recrystallization by increasing the driving force through the large stored elastic energy^24^, and hence may contribute to the increased recrystallized grain density of Cryo samples relative to the RT samples in (Fig. 4).

While both rolling conditions lead to a significant increase in the yield and ultimate tensile strengths compared to that of as-received condition, Cryorolling resulted in the highest increase (Fig. 1). These differences may be attributed to the differences in their dynamic recovery and recrystallization behaviours. Dynamic recovery could be observed under both RT and Cryogenic conditions, as revealed in the EBSD maps. While only limited partial dynamic recrystallization could be observed in RT samples (Fig. 4), the recrystallization is it is relatively suppressed in samples rolled at Cryorolling temperatures. It may be worth noting here that the Cryorolled samples did not develop any detectable amount of martensite within the detectable range of the XRD measurements. This is consistent with the report, that martensite could not be observed in a UNS S31000 even at −269 °C. The M_d30_ temperature, which was estimated using the empirical expression described below from Angel *et al*. (Eq. 1) is −168 °C^25^, Nohara *et al*. (Eq. 2) determined a value of −390 °C and Masaharu Hatano *et al*. (Eq. 3) determined a value of −393 °C^26,27^.

1 $$\begin{array}{cc}Md(^\circ C) & =\,413-13.7( \% C)-9.5( \% Ni)-8.2( \% Mn)-9.2( \% Si)-18.5( \% Mo)\\  & -\,462( \% C+ \% N)\end{array}$$

2 $$\begin{array}{ccc}Md(^\circ C) & = & 551-13.7( \% Cr)-29( \% Ni+ \% Cu)-8.1( \% Mn)-9.2( \% Si)\\  &  & -18.5( \% Mo)-462( \% C+ \% N)-68( \% Nb)-142(GS-8)\end{array}$$

3 $$\begin{array}{ccc}Md(^\circ C) & = & 551-462( \% C+ \% N)-9.2( \% Si)-8.1( \% Mn)-13.7( \% Cr)\\  &  & -29( \% Ni+ \% Cu)-18.2( \% Mo)\end{array}$$

It can be inferred that formation of martensite was unlikely as the rolling has taken place at −196 °C which is much higher than the calculated temperatures from the above mentioned empirical equations. Hence, the difference in the strengths of Cryorolled and room-temperature-rolled samples can be attributed to the dislocation sub structures which are dictated by SFE and suppression of dynamic recovery. Furthermore, the RT50 condition also exhibited a strength of more than 1000 MPa, which is higher than the expected strength value. The ductility, as characterized by the peak strain under the rolled conditions, is very low for all the samples. A post-rolling flash anneal for very brief periods at high temperatures significantly restores the ductility by a couple of tens of per cent. The high temperature enables the release of the stored elastic energy through nucleation of recrystallized grains. The very short annealing periods prevented grain growth while still retaining good amount of dislocation density (Fig. 7). Similar observations were also reported in case of TWIP steels by Tewari *et al*.^28^.

## Conclusions

The effect of Cryogenic and room temperature rolling on the strength and ductility of UNS S31000 austenitic stainless steel was investigated for 50% and 75% thickness reductions. A five-fold enhancement in the strength is observed under Cryorolled conditions while room temperature rolling has resulted in a four-fold increase. Extensive microstructural investigations using transmission electron microscopy and electron back scattered diffraction methods revealed the development of deformation nano-twins and dislocation cell walls, leading to a strength enhancement. While rolling resulted in severe reduction in ductility, flash annealing treatment between 800 °C and 850 °C for approximately two minutes has restored the ductility significantly, up to as high as 30%.

## Data Availability

The raw/processed data required to reproduce these findings cannot be shared at this time as the data also form part of an ongoing study. However, this can be provided based on requests from individuals.
